# Supplementary material for: LPS-Induced Coagulation and Neuronal Damage in a Mice Model Is Attenuated by Enoxaparin
Source: Int J Mol Sci. 2022 Sep 9;23(18):10472. doi: 10.3390/ijms231810472 (PMC9499496; doi:10.3390/ijms231810472)
Supplement: Supplementary file 1 [file ijms-23-10472-s001.zip › ijms-1872261-supplementary.pdf]

Supplementary Figures

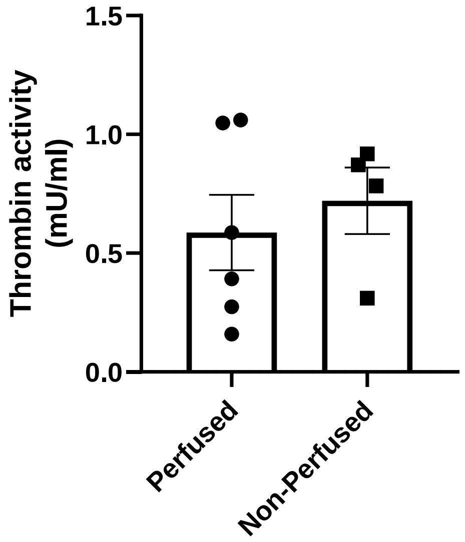

Figure S1. Cardiac perfusion does not significantly affect the thrombin activity measurements in the mice hippocampus. Thrombin activity was measured in the hippocampus derived from mice with cardiac perfusion (N = 6) and without cardiac perfusion (N = 4). Results are presented as mean ± SEM.

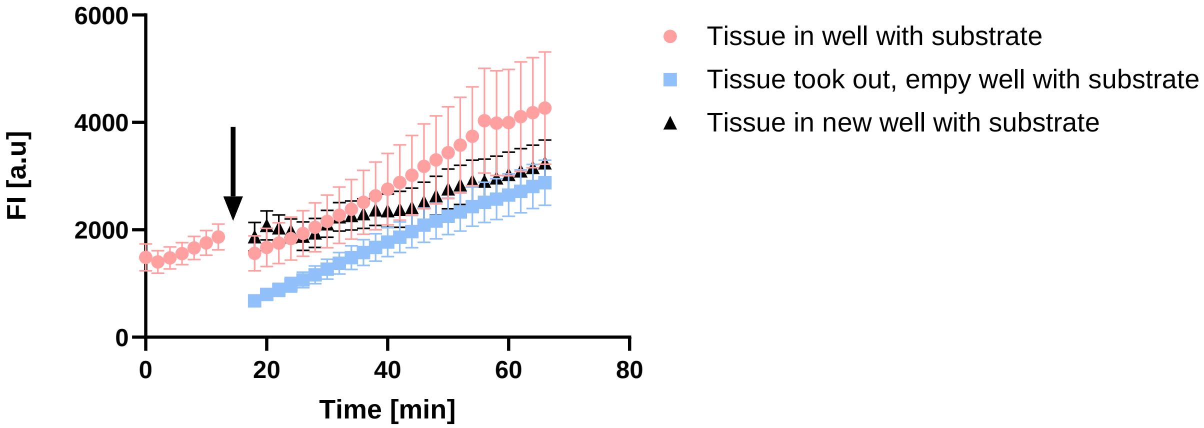

Figure S2. Thrombin activity measurements in the presence and absence of brain tissue in the well. 10 samples were placed in the wells and measured for 15 minutes (Pink circles until the time indicated by the horizontal arrow). Following these 5 samples were moved to new wells and the substrate added (Black triangles), measurements were continued in the well without the samples (Blue squares), and measurements were continued if the other 5 samples without removing the tissue (Pink circles) Results are presented as mean ± SEM.
